# A lightweight attribute-based signcryption scheme based on cloud-fog assisted in smart healthcare

**DOI:** 10.1371/journal.pone.0297002

**Published:** 2024-01-30

**Authors:** Yanzhong Sun, Xiaoni Du, Shufen Niu, Siwei Zhou

**Affiliations:** 1 College of Mathematics and Statistics, Northwest Normal University, Lanzhou, China; 2 Key Laboratory of Cryptography and Data Analytics, Northwest Normal University, Lanzhou, China; 3 Gansu Provincial Research Center for Basic Disciplines of Mathematics and Statistics, Northwest Normal University, Lanzhou, China; 4 College of Computer Science and Engineering, Northwest Normal University, Lanzhou, China; University College of Engineering Tindivanam, INDIA

## Abstract

In the environment of big data of the Internet of Things, smart healthcare is developed in combination with cloud computing. However, with the generation of massive data in smart healthcare systems and the need for real-time data processing, traditional cloud computing is no longer suitable for resources-constrained devices in the Internet of Things. In order to address this issue, we combine the advantages of fog computing and propose a cloud-fog assisted attribute-based signcryption for smart healthcare. In the constructed “cloud-fog-terminal” three-layer model, before the patient (data owner)signcryption, it first offloads some heavy computation burden to fog nodes and the doctor (data user) also outsources some complicated operations to fog nodes before unsigncryption by providing a blinded private key, which greatly reduces the calculation overhead of resource-constrained devices of patient and doctor, improves the calculation efficiency. Thus it implements a lightweight signcryption algorithm. Security analysis confirms that the proposed scheme achieves indistinguishability under chosen ciphertext attack and existential unforgeability under chosen message attack if the computational bilinear Diffie-Hellman problem and the decisional bilinear Diffie-Hellman problem holds. Furthermore, performance analysis demonstrates that our new scheme has less computational overhead for both doctors and patients, so it offers higher computational efficiency and is well-suited for application scenarios of smart healthcare.

## Introduction

Smart healthcare has emerged from the rapid advancement of the Internet of Things (IoT) [1]. By leveraging advanced techniques such as big data, artificial intelligence, cloud computing and IoT, smart healthcare has facilitated the automation, informatization, and intelligence of medical services [2], it achieves the intelligence of healthcare capabilities, improves healthcare efficiency, and optimises patient experience services. Traditional paper-based Personal Health Records (PHR) have been replaced by electronic PHR [3], resulting in a massive influx of data and increased demands for device mobility and real-time processing. These challenges have posed significant limitations to traditional cloud computing.

Owing to the above problems, fog computing has been introduced [4]. Fog computing provides a solution that is better suited to mobile IoT and a wide range of mobile applications by extending cloud computing to the network edge, closer to the end-user. With the exponential growth of IoT devices, cloud computing has faced the challenge of processing hundreds of millions of massive data. Fog computing, with its powerful computing capabilities, offers low latency and support for end user mobility, enabling the processing of data with low computing requirements. As a result, certain computing tasks is delegated to fog nodes by nearby end-users, reducing the computational burden for users, significantly improving the efficiency, and satisfies the requirements for real-time processing of mobile applications [5].

In the context of the smart health system, fog computing is positioned closer to the end-user, handling a significant amount of sensitive patient data that ultimately gets uploaded to the medical cloud for storage. Given the semi-trusted nature of the medical cloud, data processing is necessary prior to uploading to safeguard the integrity and privacy of the patient. To address this, the attribute-based cryptosystem offers enhanced privacy and security for patient data. It enables one-to-many access control with fine-grained, distinguishing itself from the traditional identity-based cryptosystem. The attribute-based cryptosystem was originated by the fuzzy identity-based cryptosystem proposed by Sahai et al. [6], which introduces the concept of attributes into the cryptosystem. This idea has found widespread application in smart health scenarios. Li et al. [7] presented an online/offline attribute-based encryption algorithm, offloading a portion of the decryption computation to ensure computational efficiency of IoT terminal devices. Similarly, Zhang et al. [8] provided a lightweight attribute-based encryption algorithm based on the smart health system, leveraging the concept of outsourcing. While traditional attribute-based cryptographic technology achieves fine-grained access control, it incurs a relatively high computational overhead. To mitigate this, Zhong et al. [9] introduced an edge-assisted attribute-based encryption algorithm that offloads part of encryption operations as well as decryption operations to edge nodes, reducing the computational burden on resource-constrained IoT devices. Drawing parallels to edge computing, fog computing also extends computational capabilities and data analysis functions to the network edge, forming a three-layer model known as “cloud-fog-devices”. This model effectively addresses the latency issue associated with cloud computing. Several studies [10–14] have explored the application of fog computing to attribute-based encryption algorithms, outsourcing a majority of the decryption operations to fog nodes, thereby significantly easing the computational load on end users. In addition, in order to ensure the integrity of the data or the legitimacy of the identity of the receiver, the message to be encrypted is usually signed and the identity is authenticated by data owner [15–17].

The traditional idea of “encrypt first and then sign” has high computational overhead and communication cost. In view of this problem, the concept and scheme of signcryption are proposed for the first time in [18], which guarantees the confidentiality and unforgerability of messages. Attribute-based signcryption has been extensively researched in recent years by various scholars [19–22], enhancing the suitability and computational efficiency of attribute-based signcryption algorithms in real-world cloud storage environments. In the context of accessing Personal Health Records (PHR), references [23, 24] propose two efficient attribute-based signcryption for multi-authority. Similarly, Liu et al. [25] present an attribute-based signcryption algorithm based on PHR, facilitating fine-grained data access control. For resource-constrained terminal devices in the IoT [26, 27], adopt a strategy of outsourcing the decryption process to edge servers, effectively reducing user-end computing overhead. Additionally [26], supports the update of access policies.

### Motivation and contributions

**Fig 1:**
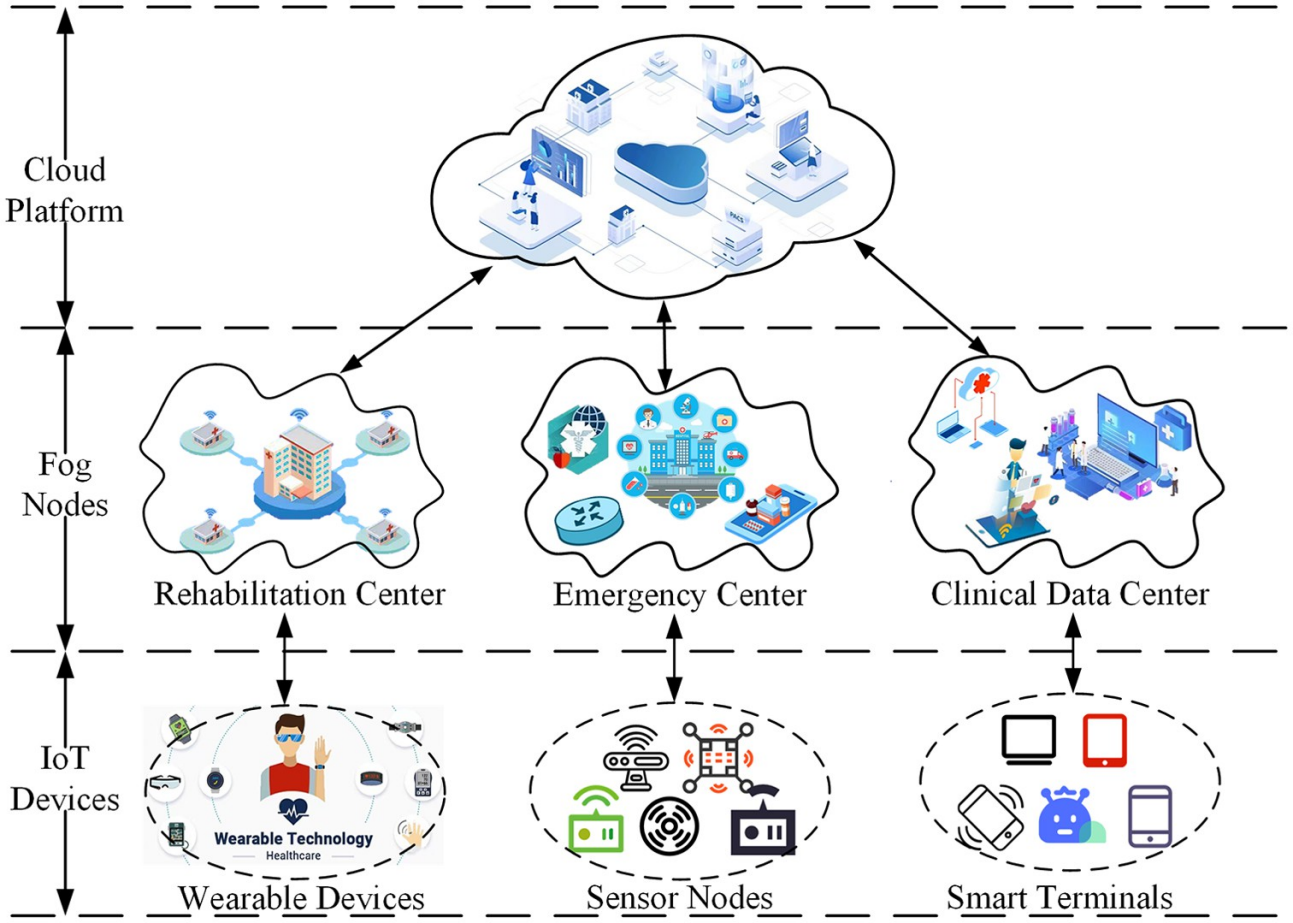
Cloud-fog-terminal three-layer structure.

Combine the advantages of cloud-fog assistance, we present a lightweight attribute-based signcryption scheme for smart healthcare. Our approach leverages the three-layer model structure known as “cloud-fog-terminal” (illustrated in Fig 1) to establish a connection between the smart health system and the medical cloud. By employing fog computing as an intermediary bridge, we exploit its low latency, location-awareness, and powerful computing capabilities to ensure secure data transmission in terms of confidentiality. Additionally, some complicated operations in the signcryption and unsigncryption processes are offloaded to the nearby fog nodes, thereby reducing the computational overhead on data users, which proves particularly advantageous for resource-constrained terminal devices in smart healthcare.

In comparison to existing schemes, our work makes noteworthy contributions in the following:

- Fog computing is structured as an intermediary link between the smart healthcare system and the medical cloud, making it easier for the fog nodes to collect and process data as they are spread around the data users. For the smart health system there are a huge number of IoT devices need to access the Internet through wireless, and have high requirements for mobility. Therefore, fog computing is more suitable for smart medical scenarios.
- In the scheme, the patient (data owner) delegates the computationally intensive tasks to the fog nodes during signcryption. Similarly, the doctor (data user) also offloads some heavy computing burden to the fog nodes by providing a blinded private key before performing unsigncryption. Specifically, in the signcryption and unsigncryption phase, the fog nodes are responsible for undertaking most of the complicated computations, such as the pairing operation. As a result, the client only needs to perform a simple multiplication operation in the process of user unsigncryption, which significantly reduces the computation burden for all client.
- The newly proposed scheme provides several advantages in terms of computational and communication overheads, as demonstrated through theoretical analysis and numerical simulation. The incorporation of fog computing significantly enhances the computational efficiency of data owner signcryption and data receiver unsigncryption in comparison to other schemes. This discrepancy becomes more pronounced as the number of attributes increases. Consequently, the proposed scheme is not only effective and practical, but also highly suitable for resource-constrained devices in the practical application of smart healthcare.

### Organization

The subsequent sections are structured as follows. Section 2 presents various fundamental concepts such as the access tree and hardness assumption. Subsequently, Section 3 is an overview of the system model, algorithm formal definition, and the system security model. Following that, Section 4 presents the construction of the scheme in detail. Section 5 is dedicated to a detailed security analysis of our scheme, while Section 6 concentrates on analysing the efficiency of the system. Lastly, Section 7 proposes the conclusion of the article.

## Preliminaries

### Access tree [7]

Let T be an access structure tree and *x* be a node in T.

(1). If *x* is a non-leaf node, then it stands for a threshold (“AND” or “OR”) gate, which can be described by (*k*_*x*_, *num*_*x*_), where *num*_*x*_ is the number of child nodes of *x* and *k*_*x*_ is its threshold value, 1 ≤ *k*_*x*_ ≤ *num*_*x*_. When *k*_*x*_ = 1, *x* is an “OR” gate; when *k*_*x*_ = *num*_*x*_, it is an “AND” gate.

(2). If *x* is a leaf node, then it stands for a attribute and described by a threshold value *k*_*x*_ = 1.

Denote the parent node of *x* in T as *parent*(*x*), and the attributes associated with the leaf node *x* in T as *att*(*x*). Each children of node *x* are numbered in a sequence from 1 to *num*_*x*_, the function *index*(*x*) denotes the index of *x* among its siblings.

Let $T_{r}$ be a access tree with root node *r*, $T_{x}$ be the subtree of $T_{r}$ rooted at *x*. If the attribute set *S* satisfies the subtree $T_{x}$, we denote it as $T_{x}\left(S\right)=1$. Furthermore, $T_{x}\left(S\right)$ can be calculated recursively by: if *x* is a non-leaf node, then $T_{x}\left(S\right)=1$ iff at least *k*_*x*_ child nodes return 1 and if *x* is a leaf node, $T_{x}\left(S\right)=1$ iff *att*(*x*) ∈ *S*.

### Bilinear pairing map [28, 29]

Let *G*_0_ and *G*_1_ be two cyclic groups with the prime order *p*, and *g* is a generator of *G*_0_. Then the bilinear map *e*: *G*_0_ × *G*_0_ → *G*_1_ can be defined as follows:

**Bilinearity:** For all $a,b\in Z_{p}^{*}$, *e*(*g*^*a*^, *g*^*b*^) = *e*(*g*, *g*)^*ab*^.

**Non-degeneracy:** *e*(*g*, *g*)≠1.

**Computability:** For all *a*, *b* ∈ *G*_0_, there is an efficient algorithm to compute the map *e*(*a*, *b*).

### Hardness assumption

#### DBDH assumption [22]

Given an cyclic group *G*_0_ with order *p*, a generator *g* of *G*_0_, a bilinear mapping *e*: *G*_0_ × *G*_0_ → *G*_1_ and $a,b,c\in Z_{p}^{*}$. The Decisional Bilinear Diffie-Hellman (CBDH) hardness assumption states that any Probabilistic Polynomial-Time (PPT) algorithm cannot decision *e*(*g*, *g*)^*abc*^ ∈ *G*_1_ and a random *Z* ∈ *G*_1_ from a given triple $\left(A=g^{a},B=g^{b},C=g^{c}\right)\in G_{1}^{3}$ with a non-negligible probability.

#### CBDH assumption [19]

Given an cyclic group *G*_0_ with order *p*, a generator *g* of *G*_0_, a bilinear mapping *e*: *G*_0_ × *G*_0_ → *G*_1_ and $a,b,c\in Z_{p}^{*}$. The Computational Bilinear Diffie-Hellman (CBDH) hardness assumption states that any PPT algorithm cannot compute *e*(*g*, *g*)^*abc*^ ∈ *G*_1_ from a given triple $\left(A=g^{a},B=g^{b},C=g^{c}\right)\in G_{1}^{3}$ with a non-negligible probability.

## System and security model

This section mainly includes an overview of the construction of system model, the formal definition of the proposed scheme and the elaboration of the system security model.

### System model

**Fig 2:**
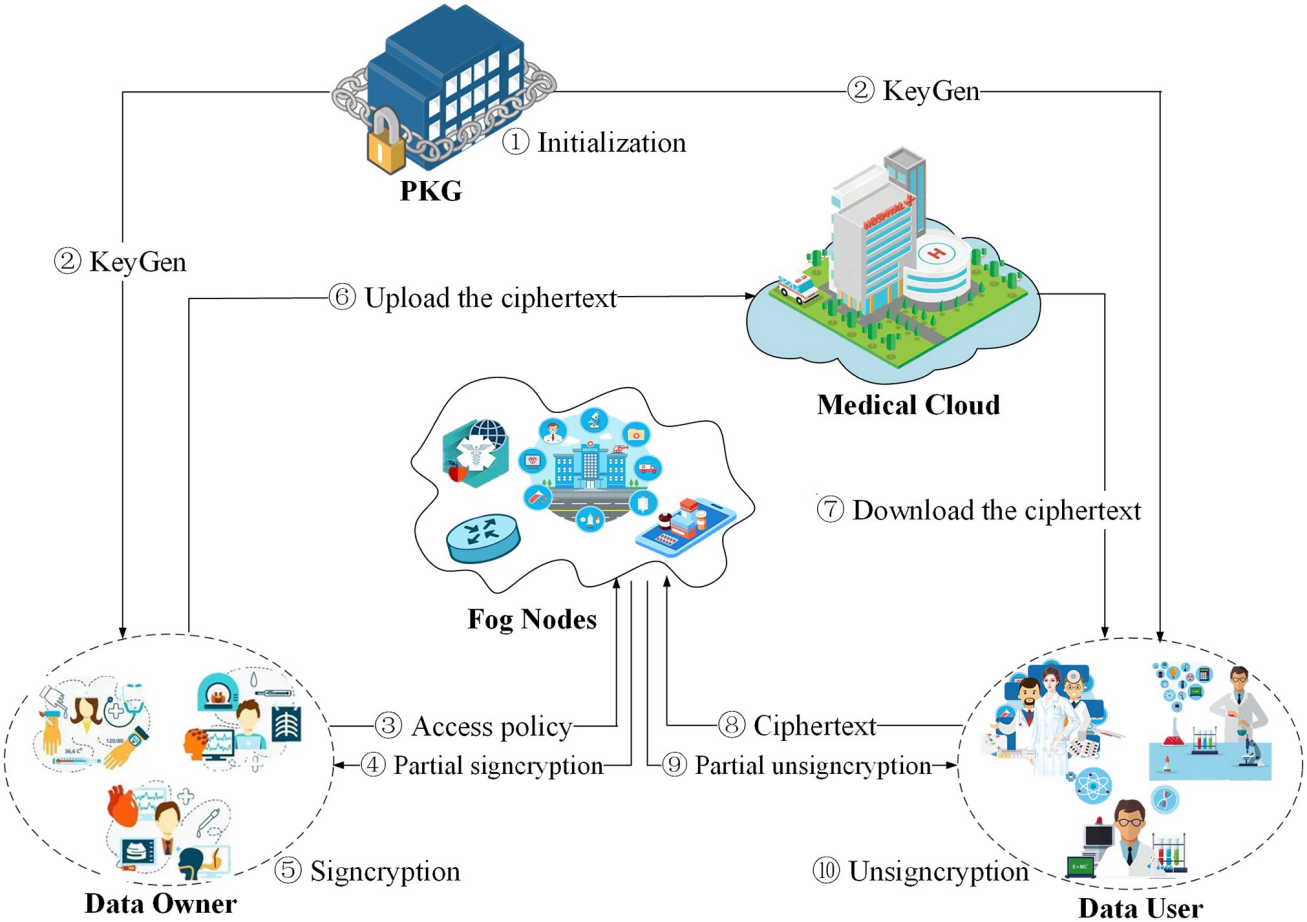
System model.

Based on fog computing, we construct the lightweight attribute-based signcryption scheme in smart healthcare. As illustrated in Fig 2, this system involves five entities, namely Private Key Generator (PKG), Medical Cloud (MC), Fog Nodes (FN), Data user (DU) and Data Owner (DO).

**Private Key Generator**: PKG initializes the system, then generates and distributes private keys to both the data owners (patients) and the data users (doctors).

**Medical Cloud**: MC refers to the server of the hospital. As a semi-trusted third party due to its powerful storage capacity, MC is mainly responsible for storing a huge number of electronic health records in the form of ciphertext provided by DO.

**Fog Nodes**: As small medical centers deployed at the network edge, FN has enough storage space and computing power to help data users with local data processing. The data owner outsources some signcryption operations to the fog node, and the data user outsources some decryption operations to the fog node, so as to reduce the computational burden of the terminal devices.

**Data Owner**: DO refers to the patients. A enormous number of patients, as the source of data in the smart health system, have ownership and control over the data. The patient defines an access structure for the data that needs to be signcrypted, specifies the scope of authorized users, and their terminal devices signcrypt the data and store the final ciphertext to the medical cloud.

**Data User**: DU refers to doctors, medical researchers, or insurance company employees who need access to patient information. When DU accesses the data, it downloads the ciphertext from the medical cloud and performs the final unsigncryption. Only the users who satisfy the access policy can decrypt the ciphertext and access the data, which realizes the fine-grained access control.

Firstly, PKG initializes the system, then generates and distributes private keys to both the data owners (patients) and the data users (doctors). The health status and personal information of the patients are collected in real time by the wearable device. The collected data is signcrypted by the fog node, producing a part of the ciphertext. Subsequently, the patients perform the final signcryption and upload the final ciphertext to the medical cloud (hospital). When data users (such as doctors, medical researchers, insurance companies, etc.) request data, the ciphertext is retrieved from the medical cloud for preliminary unsigncryption. The fog node then assists the data users in performing partial operations, ultimately allowing the users to get the required data.

### Formal definition

Now, we formalize the definition of the proposed schemes. This scheme is composed of the following six algorithms in PPT.

- *Setup*(λ, *S*)→(*pp*, *msk*): PKG executes the setup algorithm. Input system security parameter λ, output system public parameter *pp*, master key *msk*.
- *KeyGen*(*pp*, *S*, *msk*)→*SK*: PKG runs the key generation algorithm, taking as input public parameter *pp*, a attributes set *S* and master key *msk*, it generates and hands out private keys *SK* for DU.
-
$FN.Sign\left(T,SK\right)\to CT^{\prime }$ : After receiving the access structure T enacted by DO, FN takes this access policy as input and performs partial signcryption operations, and finally outputs part of the ciphertext *CT*′.
-
$DO.Sign(m,T,CT^{\prime })\to CT$ : After FN performs part of the operation, DO executes this algorithm, it takes the message *m*, the enacted access structure T and the partial ciphertext *CT*′ as the input of the algorithm, and finally generates the signature *σ* and the final ciphertext *CT*.
-
$FN.Unsign\left(CT,\bar{SK},S\right)\to \left(B,T,F\right)$ : When FN receives the blinded private key $\bar{SK}$ of DU, it performs a partial unsigncryption operation, calculates some parameters (*B*, *T*, *F*) according to the recursive formula of the access tree, then sends the parameters to DU for final unsigncryption.
- *DU*.*Unsign*(*CT*, *SK*, *S*)→*m*: DU executes this algorithm, using ciphertext *CT*, private key *SK* and attribute set *S* as the input, if DU satisfies the access structure, it will get the plaintext *m*.

### Security model

This section provides a formalization of the security of the scheme encompassing confidentiality and unforgeability, which are simulated as the following two games which are interactions between a challenger C and an adversary A within PPT.

**Game 1.** Confidentiality

**Definition 1.** The proposed attributed-based signcryption scheme is indistinguishable under chosen ciphertext attack (IND-CCA) against any adversary A possessing polynomial time capability, if the advantages of A in the following interaction are negligible:

**Initialization:** Firstly, A generates a challenge attribute set denoted as *S*. To proceed, C executes the *Setup* to returns *pp* to A. Simultaneously, it retains *msk* secretly.

**Query Phase 1:** When A initiates a series of prophecy inquiry, C responds the inquiry as described below.

- Key extraction query: in the query, when A requests a private key *SK* with the attribute set S*, C executes *KeyGen* according to S* and returns the corresponding *SK* to A.
- Signcryption query: in the query, when A queries a ciphertext for any message *m*, C proceeds by selecting an attribute set *S* from T* firstly, it then runs the key extraction query and obtain *SK*, followed by executing the signcryption algorithm to encrypt and send the corresponding ciphertext *CT* to the adversary A.
- Unsigncryption query: For any attribute set *S* and the corresponding ciphertext *CT* queried by the adversary A, The first step performed by the challenger C is to execute the key extraction query and get *SK*, it then proceeds the unsigncryption algorithm to decrypts *CT* and returns the resulting output to A.

**Challenge:** In this phase, A selects two different messages *m*_0_ ≠ *m*_1_ with equal length as the challenge message, and presents them to C to request the corresponding ciphertext. And C randomly chooses a bit *b* ∈ {0, 1}, runs the *DO*.*Sign* algorithms and *FN*.*Sign* algorithms to gnerate and return the challenge ciphertext CT* of $m_{b}^{*}$ to A.

**Query Phase 2:** A can continue issue a similar inquiry as in Query Phase 1. Any adversary can initiate a signcryption challenge on any ciphertext apart from the challenged one.

**Guess:** If A can output a bit *b*′, and *b*′ = *b*, then A wins the above game. The probablity advantage of A in the game can be defined as $Adv\left(A\right)=|pr\left[b^{\prime }=b\right]-\frac{1}{2}|$.

**Game 2.** Unforgeability

**Definition 2.** The proposed attributed-based signcryption scheme is existential unforgeability under chosen message attack (EUF-CMA) against any adversary A possessing polynomial time capability, if the advantages of adversary A in the following interaction are negligible:

**Initialization:** in this phase, A sends a attributes set *S* to C forge the ciphertext. To proceed, C executes the *Setup* to returns *pp* to A. Simultaneously, it retains *msk* secretly.

**Query Phase:** A initiates a prophecy inquiry, and C responds to the inquiry as described below.

- Key extraction query: in the query, A asks the user for the private key *SK*. After receiving attribute set S*, C runs the key generation algorithm and return the corresponding private key according to the attribute set S*.
- Signcryption query: if A wants to signcrypt message m*, C select an attribute set *S*, and *S* ∈ T*, then executes the key extraction algorithm to obtain *SK* and runs the *DO*.*Sign* algorithms and *FN*.*Sign* algorithms to obtain and return the ciphertext CT* to A.
- Unsigncryption query: let *CT* be the ciphertext with respect to attribute set *S* queried by A, C first performs the key generation algorithm to obtain *SK*, then executes the *DO*.*Unsign* algorithms and *FN*.*Unsign* algorithms, and finally sends the result to A.

**Forge:** In the phase, A outputs the forged ciphertext *CT* of the message m*, and finally if A outputs *Unsigncryption*(*CT*, *SK*, S*) = m* ≠ ⊥, then the game is won. The probablity advantage of A in winning the game can be defined as

$$\begin{array}{c} Adv_{A}^{\text{Unforgeability}}=pr\left[A\;\text{wins}\right]. \end{array}$$

### The concrete scheme

**Fig 3:**
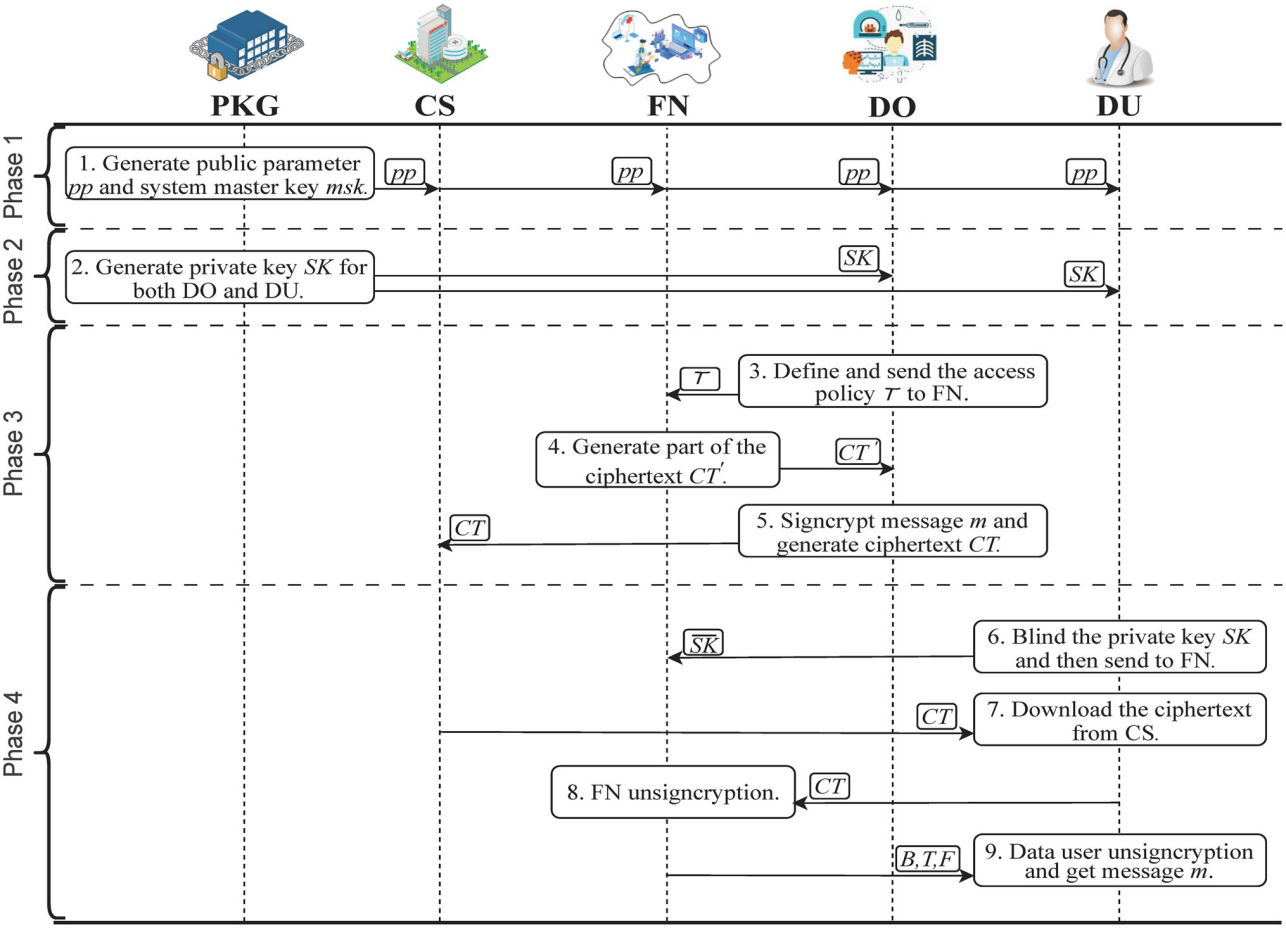
Algorithm model.

As illustrated in Fig 3, The scheme is essentially made up of four phases, system initialization, private key generation, signcryption and unsigncryption, which are described in more detail below.

**Phase 1: System initialization**

PKG performs the Setup algorithm. In the initialization phase, give security parameter λ,

- PKG selects two cyclic multiplicative groups *G*_0_ and *G*_1_ with order *p*, a generator *g* ∈ *G*_0_. Let *e*: *G*_0_ × *G*_0_ → *G*_1_ be bilinear mapping and *H*: {0, 1}* → *G*_0_ be a cryptographic hash function.
- Random selection $\alpha ,\beta \in Z_{p}^{*}$, PKG calculation *h* = *g*^*β*^. Finally, PKG publishes system parameter *pp* = (*G*_0_, *G*_1_, *g*, *e*(*g*, *g*)^*α*^, *h* = *g*^*β*^) and keep system master key *msk* = (*g*^*α*^, *β*) secretly.

**Phase 2: Private key generation**

PKG executes this algorithm, generates and issues private keys for DU.

- Randomly select $r_{1}\in Z_{p}^{*}$, generate private key $K_{1}=g^{\frac{\alpha +r_{1}}{\beta }}$.
- For all attribute *j* ∈ *S*, it selects random number $r_{j}\in Z_{p}^{*}$, and computes $K_{j}=g^{r_{1}}\cdot H{\left(j\right)}^{r_{j}}$, $K_{j}^{\prime }=g^{r_{j}}$. The private key $SK=(K_{1},K_{j},K_{j}^{\prime })$ is eventually distributed to the requested clients.

**Phase 3: Signcryption**

The signcryption algorithm includes two parts: data owner signcryption and fog node signcryption. Firstly, DO defines an access tree T and sends it to FN, it then run *FN*.*Sign* algorithm.

- FN signcryption. Fog nodes execute *FN*.*Sign* algorithm for outsource computing.
- Let *x* be a node in T, FN chooses a polynomial *q*_*x*_ and sets the degree *d*_*x*_ of *q*_*x*_ as *d*_*x*_ = *k*_*x*_ − 1, where *k*_*x*_ is the threshold value of *x*. Now, if *x* = *r* is the root node of T, then FN assigns a random number $s\in Z_{p}^{*}$ and sets *q*_*r*_(0) = *s*, if *x* ≠ *r*, then FN sets *q*_*x*_(0) = *q*_*parent*(*x*)_(*index*(*x*)).
- Let *Y* be the leaf node set in T, for ∀*y* ∈ *Y*, FN calculates *C* = *h*^*s*^ = *g*^*βs*^, *C*_*y*_ = *g*^*q*_*y*_(0)^, $C_{y}^{\prime }=H{\left(att\left(y\right)\right)}^{q_{y}\left(0\right)}$, and sends part of the ciphertext $CT^{\prime }=\left(T,C,C_{y},C_{y}^{\prime }\right)$ to DO for the next signcryption.
- DO signcryption. After receiving *CT*′ = ($T,C,C_{y},C_{y}^{\prime })$, the data owner runs the *DO*.*Sign* algorithm.
- DO first computes C* = *m* ⋅ *e*(*g*, *g*)^*αs*^, then selects a number $\mu \in Z_{p}^{*}$ randomly, and computes *σ* = *e*(*C*, *g*)^*μ*^ = *e*(*g*, *g*)^*βsμ*^, *π* = *H*(*σ*|*m*), and $\Psi =g^{\mu }\cdot D_{1}^{\pi }=g^{\mu }\cdot g^{\frac{\alpha +r_{1}}{\beta }\cdot \pi }$. Eventually, it sends the ciphertext $CT=(T,C,C_{y},C_{y}^{\prime },C^{*},W,\pi ,\Psi )$ to the medical cloud for storage.

**Phase 4: Unsigncryption**

The unsigncryption algorithm mainly includes two parts: data user unsigncryption and fog node unsigncryption. The data user selects the random number $d\in Z_{p}^{*}$, then blinds the private key *SK*, computes ${\bar{K}}_{1}={\left(K_{1}\right)}^{d}$, ${\bar{K}}_{j}={\left(K_{j}\right)}^{d}$, and ${\bar{K}}_{j}^{\prime }={\left(K_{j}^{\prime }\right)}^{d}$, and finally sends the blinded private key $\bar{SK}=\left({\bar{K}}_{1},{\bar{K}}_{j},{\bar{K}}_{j}^{\prime }\right)$ to the fog node.

- FN unsigncryption. When DU downloads the ciphertext *CT* from the medical cloud, FN first runs the *FN*.*Unsign* algorithm, which is a recursive algorithm that takes node y∈T, blinded private key $\bar{SK}$ and ciphertext *CT* as input.
- If *y* ∈ *Y* is a leaf node, and *att*(*y*) ∈ *S*, compute
  $$\begin{array}{ccc} \varphi_{y} & =\frac{e({\bar{K}}_{j},C_{y})}{e({\bar{K}}_{j}^{\prime },C_{y}^{\prime })}=\frac{e(g^{r_{1}d}\cdot H{\left(j\right)}^{r_{j}d},g^{q_{y}\left(0\right)})}{e(g^{r_{j}d},H{\left(att\left(y\right)\right)}^{q_{y}\left(0\right)})} & \\ & =\frac{e\left(g^{r_{1}d},g^{q_{y}\left(0\right)}\right)\cdot e\left(H{\left(j\right)}^{r_{j}d},g^{q_{y}\left(0\right)}\right)}{e(g^{r_{j}d},H{\left(att\left(y\right)\right)}^{q_{y}\left(0\right)})} & \\ & =e{\left(g,g\right)}^{r_{1}dq_{y}\left(0\right)}, \end{array}$$
  if *att*(*y*)∉*S*, it outputs *φ*_*y*_ = ⊥.
- If *y* is a non-leaf node, FN recursively calculates *φ*_*y*′_ of node *y*’s child node *y*′, let *S*_*y*′_ be a set of any *φ*_*y*_-sized child node {*y*′}, if *S*_*y*′_ = ∅, then *φ*_*y*′_ = *Null*; otherwise
  $$\begin{array}{ccc} \varphi_{y^{\prime }}= & \prod_{y\in S_{y}}{\varphi_{y}}^{\Delta_{i,S_{y}^{\prime }}\left(0\right)} & \\ = & \prod_{y\in S_{y}}{\left(e{\left(g,g\right)}^{r_{1}d\cdot q_{y}\left(0\right)}\right)}^{\Delta_{i,S_{y}^{\prime }}\left(0\right)} & \\ = & \prod_{y\in S_{y}}{\left(e{\left(g,g\right)}^{r_{1}d\cdot q_{parent(y)}\left(index\left(y\right)\right)}\right)}^{\Delta_{i,S_{y}^{\prime }}\left(0\right)} & \\ = & \prod_{y\in S_{y}}e{\left(g,g\right)}^{r_{1}d\cdot q_{y}\left(i\right)\cdot \Delta_{i,S_{y}^{\prime }}\left(0\right)}, & \\ = & e{\left(g,g\right)}^{r_{1}d\cdot q_{y^{\prime }}\left(0\right)}, \end{array}$$
  where $\Delta_{i,S_{y}^{\prime }}=\prod_{j\in S_{y}^{\prime },j\neq i}\frac{x-j}{i-j}$ denotes Lagrange coefficient and $S_{y}^{\prime }=\left\{index\left(y\right):y\in S_{y}\right\}$.
- If *y* = *r* is a root node, FN computes *φ*_*r*_ = *e*(*g*, *g*)^*r*_1_*ds*^.
- Finally, FN calculates
  $$\begin{array}{c} \begin{array}{cc} & B\;=e\left(C,{\bar{K}}_{1}\right)=e\left(g^{\beta s},g^{\frac{\alpha +r_{1}}{\beta }\cdot d}\right)=e{\left(g,g\right)}^{sd(\alpha +r_{1})},\\ & T\;=\frac{B}{\varphi_{r}}=\frac{e{\left(g,g\right)}^{sd(\alpha +r_{1})}}{e{\left(g,g\right)}^{r_{1}ds}}=e{\left(g,g\right)}^{sd\alpha },\\ & F\;=e\left(C,\Psi \right)=e\left(g^{\beta s},g^{\mu }\cdot g^{\frac{\alpha +r_{1}}{\beta }\cdot \pi }\right)\\ & \;\;\;=e\left(g^{\beta s},g^{\mu }\right)e\left(g^{\beta s},g^{\frac{\alpha +r_{1}}{\beta }\cdot \pi }\right)\\ & \;\;\;=e{\left(g,g\right)}^{\mu \beta s}e{\left(g,g\right)}^{s\pi (\alpha +r_{1})}, \end{array} \end{array}$$
  then sends (*B*, *T*, *F*) to DU for the next unsigncryption.
- DU unsigncryption. DU performs the final unsigncryption by receiving the parameters sent by FN.
- Firstly, DU computes
  $$\begin{array}{c} T^{\prime }=T^{d^{-1}}=e{\left(g,g\right)}^{s\alpha },\;B^{\prime }=B^{d^{-1}}=e{\left(g,g\right)}^{s(\alpha +r_{1})}. \end{array}$$
- Then decrypt message:
  $$\begin{array}{c} \begin{array}{c} m^{\prime }=\frac{C^{*}}{T^{\prime }}=\frac{m\cdot e{\left(g,g\right)}^{\alpha s}}{e{\left(g,g\right)}^{s\cdot \alpha }}=m. \end{array} \end{array}$$
- Finally verify the correctness of the signature by the equation:
  $$\begin{array}{c} \begin{array}{c} \sigma^{\prime }=\frac{F}{B^{\prime \pi }}=\frac{e{\left(g,g\right)}^{\beta s\mu }\cdot e{\left(g,g\right)}^{s\pi (\alpha +r_{1})}}{e{\left(g,g\right)}^{s\pi (\alpha +r_{1})}}=e{\left(g,g\right)}^{\beta s\mu }=\sigma . \end{array} \end{array}$$

## Security analysis

Based on the DBDH and CBDH hardness assumption, this section provides a thorough security analysis process of the scheme. Specifically, our primary focus is on ensuring confidentiality and unforgeability.

**Theorem 1.** The proposed attribute-based signcryption scheme satisfies IND-CCA security if the DBDH hardness assumption holds.

**Proof.** If there exists an adversary A who can win in Game 1 with a non-negligible probability advantage *ε* within PPT *t*, which means there is an algorithm can effectively break the confidentiality of the scheme with probability *ε* within PPT. With the help of the adversary A, we construct an algorithm B to decision DBDH problem associated Challenger C.

Given a tuple (*A* = *g*^*a*^, *B* = *g*^*b*^, *C* = *g*^*c*^), $a,b,c\in Z_{p}^{*}$ as an example of the DBDH assumption, B tries to decision *e*(*g*, *g*)^*abc*^ ∈ *G*_1_ and *Z* ∈ *G*_1_. Let *CT* be a challenge ciphertext, C* be part of the ciphertext randomly selected from *m*_0_*e*(*g*, *g*)^*αs*^ or *m*_1_*e*(*g*, *g*)^*αs*^. Let *θ* = *αs*, $\theta \in Z_{p}^{*}$, *CT* be *e*(*g*, *g*)^*αs*^ or *e*(*g*, *g*)^*θ*^. B must distinguish *m*_0_*e*(*g*, *g*)^*αs*^ and *m*_1_*e*(*g*, *g*)^*αs*^, and needs to make a distinction between *e*(*g*, *g*)^*αs*^ and *e*(*g*, *g*)^*θ*^.

**Initialization:** Adversary A first selects and sends attribute set S* to B as a target attribute set, B randomly selecting $\alpha ,\beta \in Z_{p}^{*}$. If *β* = 0, then the Setup algorithm terminates, otherwise B runs the Setup algorithm and generate the public parameter *pp*. And then B sends (*h* = *g*^*β*^, *e*(*g*, *g*)^*α*^) to A. If A queries the value of *H*, then B randomly selects $t_{j}\in Z_{p}^{*}$ and answers $g^{t_{j}}$.

**Query phase 1:** During this phase, adversary makes several inquiries.

- Key extraction query: For target attribute set S*, if A multiple requests the private key *SK*, B randomly chooses $r_{1}\in Z_{p}^{*}$, let $K_{1}=g^{\frac{\alpha +r_{1}}{\beta }}$. For attributes *j* ∈ *S*, B chooses $r_{j}\in Z_{p}^{*}$ randomly and computes $K_{j}=g^{r_{1}}\cdot H{\left(j\right)}^{r_{j}}$, $K_{j}^{\prime }=g^{r_{j}}$. Eventually, it returns the private key as $SK=(K_{1},K_{j},K_{j}^{\prime }),\;\forall j\in S$ to the adversary A. When A queries the i-th key extraction challenge with attribute set *S*_*i*_, algorithm B chooses another random number $r^{\left(i\right)}\in Z_{p}^{*}$, computes $K_{1}=g^{\frac{\alpha +r^{\left(i\right)}}{\beta }}$, and for ∀*j* ∈ *S*_*i*_, it computes $K_{j}=g^{r^{\left(i\right)}}\cdot {t_{j}}^{r_{j}^{\left(i\right)}}$, $K_{j}^{\prime }=g^{r_{j}^{\left(i\right)}}$, and sends this result to adversary A.
- Signcryption query: Signcryption query: Adversary A requests to signcrypt message m*. For each message, B runs the key generation algorithm to obtain the private key, then executes the signcryption algorithm to obtain the ciphertext CT*, and returns to A.
- Unsigncryption query: At this stage, adversary A issues a unsigncryption request for ciphertext *CT*. First, B verifies whether *C* = C* is true, because *C* = *g*^*θ*^ is random in the eyes of adversary A, and the probability of this ciphertext is at most 1/*p*. If the equation holds, B terminates the algorithm, otherwise the following steps are performed:
- If S* = 0, the access control tree T for unsigncrypting is not satisfied. B execute the key generation algorithm to get the private key, then run the unsigncryption algorithm, and return the result to A.
- If S* = 1, it satisfies the access control tree T for unsigncryption. B first verify signature *σ*′ = *e*(*C*, Ψ)/*B*′^*π*^, if incorrect, output ⊥. Otherwise, it uses $e\left({\bar{K}}_{j},C_{y}\right)/e\left({\bar{K}}_{j}^{\prime },{C_{y}}^{\prime }\right)$ to determine $e{\left(g,g\right)}^{r^{\left(i\right)}\cdot q_{y}(0)\cdot d}$.
- If the access structure satisfies the attribute set, let $B=FN.Unsign(CT,SK,r)=e{\left(g,g\right)}^{r^{\left(i\right)}\cdot q_{y}(0)\cdot d}=e{\left(g,g\right)}^{r_{1}\cdot s\cdot d}$. Then calculate
  C*/C*B′/B′φR′φR′B′/B′φR′φR′=m·e(g,g)αs/m·e(g,g)αse(g,g)s(α+r1)/e(g,g)s(α+r1)e(g,g)r1se(g,g)r1se(g,g)s(α+r1)/e(g,g)s(α+r1)e(g,g)r1se(g,g)r1s=m.
  Finally, the recovered message *m* is sent to A.

**Challenge:** In this phase, A selects two different messages *m*_0_ ≠ *m*_1_ with equal length, B randomly chooses *b* ∈ {0, 1}, and signcrypts message $m_{b}^{*}$ based on challenge attribute set S*. The process for generating challenge ciphertext CT* is the following: first, B randomly chooses $s\in Z_{p}^{*}$, and uses *f*_*j*_ to recover the secret *s* or attribute *j*; calculates $C^{*}=m_{b}^{*}\cdot Z$, *C*_*j*_ = *g*^*ab*^, and $C_{j}^{\prime }=g^{t_{j}f_{j}}$; randomly selects $\varsigma \in Z_{p}^{*}$, and calculates $\sigma *=e{\left(C,g\right)}^{\varsigma }$, $\pi^{*}=H\left(\sigma^{*}|m_{b}^{*}\right)$, and $\Psi^{*}=g^{\varsigma }\cdot D_{j}^{\pi^{*}}$. Finally B sends $CT^{*}=\left(T^{*},C^{*},C_{j},C_{j}^{\prime },\pi^{*},\Psi^{*}\right)$ to A.

**Query phase 2:** For the second challenge initiated by adversary A, the reply process of B is similar to that of challenge phase 1. There is no unsigncryption challenge during this challenge phase.

**Guess:** Adversary A finally outputs a bit *b*′ and if *b*′ = *b*, then we claim that A wins the game. If *Z* = *e*(*g*, *g*)^*abc*^, then CT* is valid with an advantage of *ε*. So $pr\left[Z=e{\left(g,g\right)}^{abc}\right]=pr\left[b^{\prime }=b|Z=e{\left(g,g\right)}^{abc}\right]=\frac{1}{2}+\varepsilon$, and the probability advantage of A is $\frac{\varepsilon }{2}$.

**Theorem 2.** The proposed attribute-based signcryption scheme satisfies EUF-CMA security based the CBDH hardness assumption.

**Proof.** Suppose there is an adversary A that can win game 2 with a non-negligible advantage *ε* in the probability polynomial time *t*, then an algorithm B can be constructed with the help of adversary A. Challenger C is given (*A* = *g*^*a*^, *B* = *g*^*b*^, *C* = *g*^*c*^), $a,b,c\in Z_{p}^{*}$ as an instance in the CBDH problem, and adversary A tries to guess *e*(*g*, *g*)^*abc*^. Let *θ* = *αs* and $\theta \in Z_{p}^{*}$.

**Initialization:** Adversary A sends target attribute set S* to B, B chooses $\alpha ,\beta \in Z_{p}^{*}$ randomly. If *β* = 0, then the system Setup algorithm terminates, otherwise B run the system Setup algorithm to obtain public parameters *pp*. Then B sends *h* = *g*^*β*^ and *e*(*g*, *g*)^*α*^ to A. When A asks for the value of *H*, B randomly chooses $t_{j}\in Z_{p}^{*}$, and answers $g^{t_{j}}$.

**Query Phase:** Adversary A initiates queries in each of the following phases.

- Key extraction query: if A requests private key *SK* based on attribute set S* multiple times, B randomly selects $r_{1}\in Z_{p}^{*}$, let $K_{1}=g^{\frac{\alpha +r_{1}}{\beta }}$. For attribute *j* ∈ *S*, select $r_{j}\in Z_{p}^{*}$ at random and compute $K_{j}=g^{r_{1}}\cdot H{\left(j\right)}^{r_{j}}$ and $K_{j}^{\prime }=g^{r_{j}}$. So the private key is: $SK=(K_{1},\forall j\in S:K_{j},K_{j}^{\prime })$, and then B returns *SK* to adversary A. When A sends the i-th key extraction to ask for the attribute set *S*_*i*_, B selects $r^{\left(i\right)}\in Z_{p}^{*}$ randomly, computs $K_{1}=g^{\frac{\alpha +r^{\left(i\right)}}{\beta }}$, and for ∀*j* ∈ *S*_*i*_, computs $K_{j}=g^{r^{\left(i\right)}}\cdot {t_{j}}^{r_{j}^{\left(i\right)}}$, $K_{j}^{\prime }=g^{r_{j}^{\left(i\right)}}$, and sends $\left(K_{1},K_{j},K_{j}^{\prime }\right)$ to A.
- Signcryption query: B sets access control policy $T^{*}$ for authorized attribute set S*. If the challenged attribute set S* does not satisfy access control policy $T^{*}$, then B can obtain the private key through the key generation algorithm. Then run the signcryption algorithm to send ciphertext *CT* to A. Assuming that attribute set S* satisfies access control policy $T^{*}$, B selects $b\in Z_{p}^{*}$ randomly, and then use *b* to recover secret *s* or attribute *j*. B randomly selects $\theta \in Z_{p}^{*}$, run the signcryption algorithm, and calculate $C^{*}=m^{*}\cdot e{\left(g,g\right)}^{\theta^{\prime }}$, *C*_*j*_ = *g*^*ab*^, $C_{j}^{\prime }={t_{j}}^{f_{j}}$. Select $c\in Z_{p}^{*}$ at random, calculate *σ* = *e*(*C*, *g*)^*c*^, *π* = *H*(*σ*|*m*), $\Psi =g^{c}\cdot D_{1}^{\pi }$. Finally, B sends ciphertext $CT=(T,C^{*},C_{j},C_{j}^{\prime },\pi ,\Psi )$ to A.
- Unsigncryption query: Adversary A initiates a unsigncryption query for ciphertext *CT* based on attribute set S*. B run the key generation algorithm to obtain private key *SK*, perform the unsigncryption algorithm, and send result *m* or ⊥ to A.

**Forgery:** After A outputs a valid forged ciphertext $CT^{*}=\left(T^{*},C^{*},C_{j},C_{j}^{\prime },\pi^{*},\Psi^{*}\right)$. The challenger C solves the CBDH assumption as follows. Since CT* is a valid ciphertext for m*, which means it can pass the verification equation, then there are *C*_*j*_ = *g*^*ab*^, *π* = *H*(*σ*|*m*), $\Psi =g^{c}\cdot D_{1}^{\pi }$, *σ* = *e*(*C*, *g*)^*c*^. Adversary A outputs a fake verification $\sigma^{*}=\frac{e(C,\Psi )}{B^{\prime \pi }}$ and $S^{*}\in T^{*}$ using attribute set S*. If S* ≠ 0, then B terminates the algorithm, verifying that equation σ* holds. If the algorithm *Unsigncrypt*(*CT*, *SK*, *S*) = m* ≠ ⊥ then A wins the game. The advantages of solving the CBDH problem are:

$$\begin{array}{c} \begin{array}{c} \begin{array}{cc} Adv_{B}^{CBDH} & =pr[B\left(g^{a},g^{b},g^{c}\right)=e{\left(g,g\right)}^{abc}]\\ & =pr[A\;\text{wins}\;\text{the}\;\text{Unforgeability}\;\text{game}]\\ & =Adv_{A}^{\text{Unforgeability}}>\varepsilon . \end{array} \end{array} \end{array}$$

## Performance comparison

This section analyzes the advantages and disadvantages of our new scheme in relation to communication and computational capabilities. Compared with some existing attribute-based signcryption schemes [22–25, 27], the capabilities of our new scheme is improved significantly. For convenience, the symbols used in this section are first summarised in Table 1.

**Table 1:** Notations.

Notations	Description
*T*_*e*_	Time of exponential operation
*T*_*h*_	Time of hash operation
*T*_*p*_	Time of pairing operation
*n*	Number of attributes of users
|*G*_0_|(|*G*_1_|, |*Z*_*r*_|)	Bit length of elements in *G*_0_(*G*_1_, *Z*_*r*_)

### Communication overhead

The viability of a scheme, particularly for resource-constrained IoT devices, heavily relies on communication overhead. The comparison between our new scheme and the schemes of [22–25, 27] regarding communication overhead is presented in Table 2. The communication overhead of various stages, namely system initialization, key generation, signcryption, and unsigncryption, is primarily taken into consideration. When it comes to the key generation stage, the communication overhead of each scheme varies based on the number of attributes. However, our scheme stands out with the smallest overhead. By employing outsourcing techniques, the communication overhead of data users in the signcryption phase becomes independent of the number of attributes. This reduction in storage burden on the local side is of utmost importance for resource-constrained IoT devices.

**Table 2:** Communication overhead.

Scheme	Setup	Keygen	Signcryption	Unsigncryption
Hong [22]	(1 + *n*)|*G*_1_|+ 2|*Z*_*r*_|	(2 + 2*n*)|*G*_0_| + 2|*G*_1_|	(3 + *n*)|*G*_0_| + (1 + 2*n*)|*G*_1_|	(3 + *n*)|*G*_1_|
Zhao [24]	*n*|*G*_1_|	(4 + 4*n*)|*G*_0_|	(2 + 3*n*)|*G*_0_|	2*n*|*G*_0_| + 3|*G*_1_| + 3*n*|*Z*_*r*_|
Liu [25]	(3 + 2*n*)|*G*_0_| + |*G*_1_|	(4 + 3*n*)|*G*_0_|	(5 + 4*n*)|*G*_0_|	*n*|*G*_0_| + (5 + 4*n*)|*G*_1_| + *n*|*Z*_*r*_|
Ruan [23]	(5 + *n*)|*G*_0_| + |*G*_1_|	(4 + 2*n*)|*G*_0_|	6|*G*_0_| + 2|*Z*_*r*_|	(3 + *n*)|*G*_0_| + *n*|*G*_1_| + 2*n*|*Z*_*r*_|
Yu [27]	(7 + 3*n*)|*G*_0_| + |*G*_1_|	(6 + 3*n*)|*G*_0_|	(7 + *n*)|*G*_0_|	(6 + 2*n*)|*G*_0_| + 2|*G*_1_| + *n*|*Z*_*r*_|
Ours	2|*G*_0_| + |*G*_1_| + 2|*Z*_*r*_|	(3 + *n*)|*G*_0_|	2|*G*_0_| + 2|*G*_1_| + 2|*Z*_*r*_|	(3 + 2*n*)|*G*_0_|

### Computational overhead

For the computational overhead, we will compare it from the aspects of theoretical analysis and numerical experiments.

#### Theoretical analysis

We primarily consider the computational burden of various stages, including system initialization, key generation, signcryption and unsigncryption. To provide a clear comparison, Table 3 provides a comparison of the computational cost between our proposed scheme and the schemes referenced in [22–25, 27]. This comparison is based on the fact that addition and multiplication operations are significantly less computationally expensive than exponentiation, bilinear pairing, and hash operations. Thus, our main point of comparison revolves around the number of exponential operations, bilinear pairing operations, and hash operations across different schemes.

**Table 3:** Computation overhead.

Scheme	Setup	Keygen	DO.Signcryption	DU.Unsigncryption
Hong [22]	*T*_*e*_ + (1 + *n*)*T*_*p*_	(2 + 2*n*)*T*_*e*_ + 2*T*_*p*_	(1 + *n*)*T*_*e*_ + (1 + 2*n*)*T*_*p*_ + *T*_*h*_	(1 + *n*)*T*_*e*_ + (1 + 2*n*)*T*_*p*_
Zhao [24]	2*nT*_*p*_	(2 + 4*n*)*T*_*e*_	(3 + 3*n*)*T*_*e*_ + 2*T*_*h*_	(1 + *n*)*T*_*e*_ + 6*T*_*p*_ + 3*T*_*h*_
Liu [25]	(3 + 2*n*)*T*_*e*_ + *T*_*p*_	(4 + 2*n*)*T*_*e*_	(5 + 4*n*)*T*_*e*_	(2 + *n*)*T*_*e*_ + (1 + 2*n*)*T*_*p*_
Ruan [23]	*T*_*e*_ + *T*_*p*_	(6 + 2*n*)*T*_*e*_	(8 + 5*n*)*T*_*e*_ + 4*T*_*h*_	2*nT*_*e*_ + 2*T*_*p*_ + *T*_*h*_
Yu [27]	*T*_*e*_ + *T*_*p*_	(6 + 5*n*)*T*_*e*_	(15 + 3*n*)*T*_*e*_ + 3*T*_*h*_	4*T*_*e*_ + 2*T*_*h*_
Ours	2*T*_*e*_ + *T*_*p*_	(3 + *n*)*T*_*e*_ + *nT*_*h*_	2*T*_*e*_ + 2*T*_*p*_ + *T*_*h*_	3*T*_*e*_

Table 3 reveals that the computational cost of the proposed scheme remains constant during the system initialization, DO signcryption and DU unsigncryption phases, regardless of the number of attributes. However, in [22], the key generation stage involves pairing operations, resulting in higher computational costs compared to other schemes. Furthermore, the schemes in [22–25] do not utilize outsourcing computing, which means that the computational cost for users varies depending on the complexity of the access policy. On the other hand, our approach ensures a stable computational overhead for data user signcryption, with most calculations outsourced to fog nodes during the signcryption phase. This includes the high-overhead pairing operation, allowing end users to decrypt messages without engaging in pairing operations. By employing outsourcing technology, the computation burden for data owners and users is minimized, further emphasizing the lightweight nature of this scheme.

#### Numerical simulation

The numerical simulation comparison was conducted on a Linux operating system, utilizing a pairing-based cryptography library with Type-A bilinear pairing parameters [30]. The programming was implemented in C language and executed on a PC with a 2.60 GHz CPU and 8 GB RAM. Our focus was primarily on observing the time variations during the system initialization stage, key generation stage, signcryption stage, and unsigncryption stage. We performed tests by altering the number of attributes, simulating attribute values from 20 to 140.

**Fig 4:**
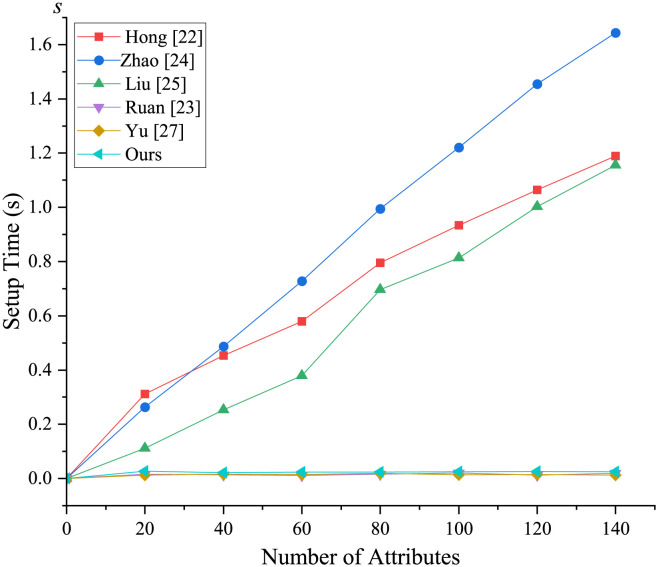
Computational overhead in Setup phase.

**Fig 5:**
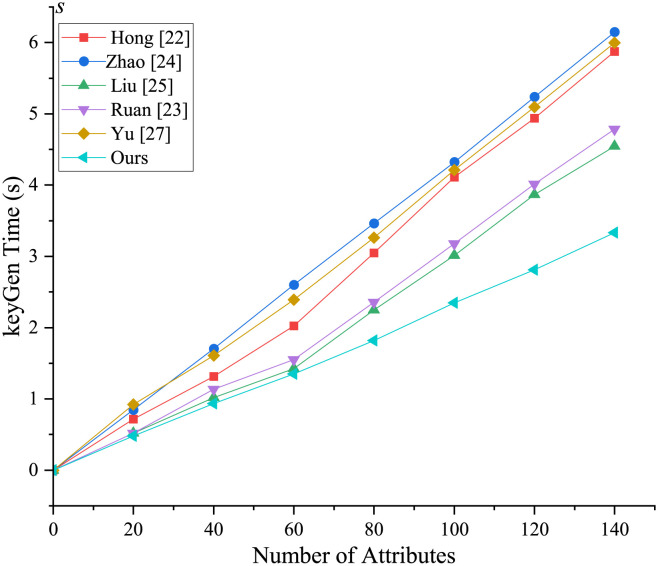
Computational overhead in KeyGen phase.

**Fig 6:**
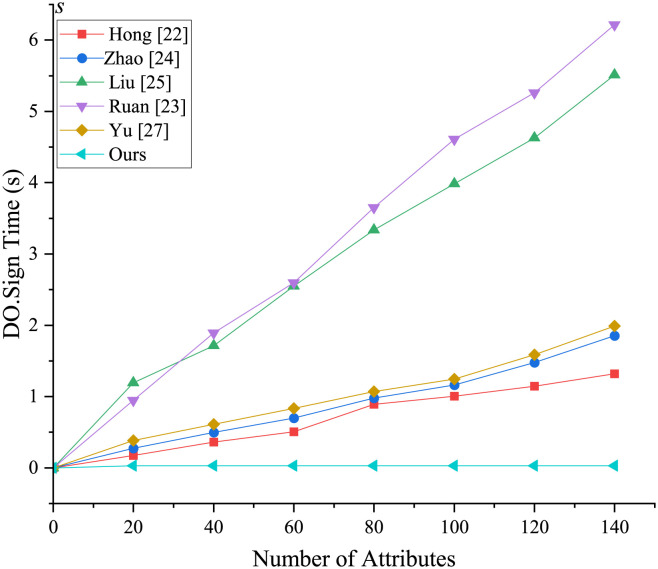
Computational overhead in DO.Sign phase.

**Fig 7:**
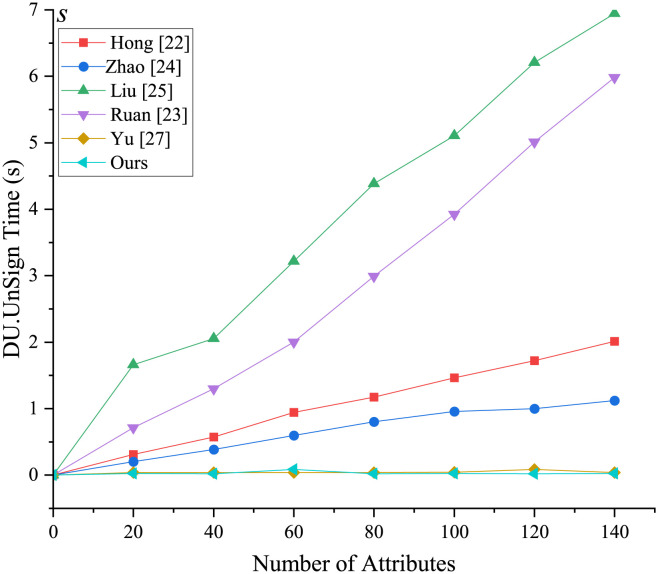
Computational overhead in DU.UnSign phase.

Fig 4 illustrates the initialization stage, where the setup algorithm is independent of attributes and follows a nearly straight line. However, the initialization stages except for [23, 27] and our scheme are attribute-dependent, resulting in an increase in setup time as the number of attributes changes. In the key generation phase (Fig 5), both the proposed scheme and the comparison scheme experience an increase in time as the number of attributes increases, but the proposed scheme performs the fastest. From Figs 6 and 7, it is evident that the running time of our new scheme in the DO signcryption and DU unsigncryption stages are optimal and attribute-independent, and thus their efficiency in terminal devices are the highest. This is due to we offload some heavy computation from the original client to the fog node, which means the scheme effectively reduces the computation of the resource-constrained devices, and improves the overall efficiency of the scheme, making it more suitable for smart healthcare scenarios. In the signcryption and unsigncryption stages, [24] demonstrates higher efficiency, but this is based on the assumption of a single attribute authority, whereas the actual scheme involves multiple attribute authorities. Therefore, if multiple attribute authorities are present, the efficiency of [24] will decrease. Through comprehensive analysis, we claim that the proposed scheme significantly enhances algorithm efficiency by partially outsourcing signcryption and unsigncryption operations to the fog node, aligning it more suitable for real-world application environments.

## Conclusion

Aiming at the generation of massive data in smart health system and the attention of patients to privacy of personal health information. We proposes a lightweight attribute-based signcryption scheme based on cloud-fog assist. In smart healthcare system, we should not only consider the basic security requirements of medical data sharing, patient privacy and confidentiality, but also consider the computing and storage capabilities of resource-constrained devices in the IoT. Therefore, fog nodes are introduced in this paper, and a three-layer model structure of “cloud-fog-terminal” is constructed, and part of the operations in the signcryption stage and the unsigncryption stage are outsourced to the fog nodes, so that the end user’s calculation cost in the signcryption stage is reduced. None pairing operation is involved, which significantly improves the computational efficiency of the scheme. Finally, under the random oracle model, the feasibility and security of the scheme are proved. Compared with the previous attribute-based signcryption scheme, our proposed scheme has better advantages in computing efficiency and is more suitable for practical smart medical application scenarios. As future work, we plan to conduct a series of simulated real-world experiences to evaluate the performance and practicality of our signcryption scheme, and design efficient a post-quantum based attribute-based signcryption scheme applicable to smart healthcare.
